# Artificial Intelligence Language Model Performance for Rapid Intraoperative Queries in Plastic Surgery: ChatGPT and the Deep Inferior Epigastric Perforator Flap

**DOI:** 10.3390/jcm13030900

**Published:** 2024-02-04

**Authors:** Connor J. Atkinson, Ishith Seth, Yi Xie, Richard J. Ross, David J. Hunter-Smith, Warren M. Rozen, Roberto Cuomo

**Affiliations:** 1 Department of Plastic and Reconstructive Surgery, Frankston Hospital, Peninsula Health, Frankston, VIC 3199, Australia; 2 Faculty of Medicine and Surgery, Monash University, Clayton, VIC 3800, Australia; 3 Faculty of Medicine and Surgery, The University of Melbourne, Parkville, VIC 3052, Australia; 4 Plastic Surgery Unit, Department of Medicine, Surgery and Neuroscience, University of Siena, 53100 Siena, Italy; roberto.cuomo2@unisi.it

**Keywords:** ChatGPT, artificial intelligence, large language model, DIEP, intraoperative, plastic surgery

## Abstract

**Background**: The integration of artificial intelligence in healthcare has led to the development of large language models that can address various medical queries, including intraoperatively. This study investigates the potential of ChatGPT in addressing intraoperative questions during the deep inferior epigastric perforator flap procedure. **Methods**: A series of six intraoperative questions specific to the DIEP flap procedure, derived from real-world clinical scenarios, were proposed to ChatGPT. A panel of four experienced board-certified plastic surgeons evaluated ChatGPT’s performance in providing accurate, relevant, and comprehensible responses. **Results**: The Likert scale demonstrated to be medically accurate, systematic in presentation, and logical when providing alternative solutions. The mean readability score of the Flesch Reading Ease Score was 28.7 (±0.8), the Flesch–Kincaid Grade Level was 12.4 (±0.5), and the Coleman–Liau Index was 14.5 (±0.5). Suitability-wise, the DISCERN score of ChatGPT was 48 (±2.5) indicating suitable and comprehensible language for experts. **Conclusions**: Generative AI tools such as ChatGPT can serve as a supplementary tool for surgeons to offer valuable insights and foster intraoperative problem-solving abilities. However, it lacks consideration of individual patient factors and surgical nuances. Nevertheless, further refinement of its training data and rigorous scrutiny under experts to ensure the accuracy and up-to-date nature of the information holds the potential for it to be utilized in the surgical field.

## 1. Introduction

The integration of artificial intelligence (AI) and machine learning (ML) technologies into the medical field has brought about remarkable advancements, particularly in the domain of clinical decision support systems [1,2,3,4]. Among these advances, large language models (LLMs) represent a cutting-edge application of AI in the realm of natural language processing. These models have garnered significant interest for their potential to augment healthcare processes, facilitate real-time communication, and provide valuable insights to healthcare professionals [3,5,6,7]. Notably, LLMs, such as Generative Pre-trained Transformer (GPT) models, demonstrate capabilities in understanding and generating human-like text, thereby facilitating real-time communication and providing valuable insights to healthcare professionals [5,6,7]. By training LLMs on extensive clinical data and medical literature, it becomes possible to develop AI systems that can support surgeons with intraoperative queries and difficulties.

The deep inferior epigastric perforator (DIEP) flap is the gold standard reconstructive option for women undergoing autologous breast cancer reconstruction [8]. It is a complex and intricate microsurgical operation involving the transfer of subcutaneous tissue and blood vessels from the abdominal region to the chest wall to reconstruct the breast mound. The procedure requires a high level of precision and technical skill, thus making it imperative for the operating surgeon to have comprehensive knowledge. The success of the DIEP flap procedure hinges on meticulous planning and execution, given its complexity and microsurgical nature. Intraoperative decision making is a critical aspect of the DIEP flap procedure, as it involves various considerations such as flap design, perforator selection, and microvascular anastomosis [9]. Advancements of innovative LLMs like ChatGPT present a promising solution to this challenge. By leveraging its ability to comprehend and generate context-specific information, ChatGPT can offer instantaneous responses to intraoperative queries. This capability is particularly valuable in scenarios where the operating surgeon needs quick access to scientific knowledge to make informed decisions. The model’s potential to bridge the gap between theoretical knowledge, as found in guidelines and research articles, and real-time surgical applications can significantly enhance the efficiency and precision of the DIEP flap procedure.

ChatGPT is an advanced natural language processing model that has demonstrated remarkable success in generating human-like responses across various domains including plastic surgery [1,2,3,4,5,6,7,8,9,10]. By harnessing the power of ML, ChatGPT can provide accurate, concise, and relevant information in a conversational manner. The integration of ChatGPT into the intraoperative environment may serve as an invaluable resource for plastic surgeons, potentially enhancing surgical outcomes and patient care. In the context of complex surgical procedures like the DIEP flap, ChatGPT’s capabilities could be harnessed to provide real-time, evidence-based answers to surgical queries. This includes offering insights on anatomical variations, suggesting procedural modifications based on patient-specific factors, and providing quick references to the latest research and clinical guidelines [1,2,3,4]. Such a tool could be pivotal in decision-making processes during surgery, potentially enhancing surgical outcomes and patient care. Moreover, ChatGPT’s ability to learn and adapt over time through continuous training and updates means that it can stay current with the latest medical advances and surgical techniques. This feature is particularly crucial in fields like plastic surgery where new techniques and research findings emerge regularly [3,4,5,6,7]. The utilization of ChatGPT in the operating room could also facilitate a more collaborative approach, allowing surgical teams to access shared knowledge bases and reduce the cognitive load on the operating surgeon, ultimately contributing to improved patient safety and care quality.

In this case study, we evaluate the performance of ChatGPT in addressing intraoperative queries related to the DIEP flap procedure. We assess the LLM’s ability to provide accurate, relevant, and timely information, as well as its overall utility in a clinical setting. By examining the role of ChatGPT in addressing intraoperative queries in plastic surgery, this study seeks to contribute to the growing body of research on AI and ML in healthcare and explore the potential for LLM to enhance surgical decision-making and patient outcomes.

## 2. Materials and Methods

**Figure 1:**
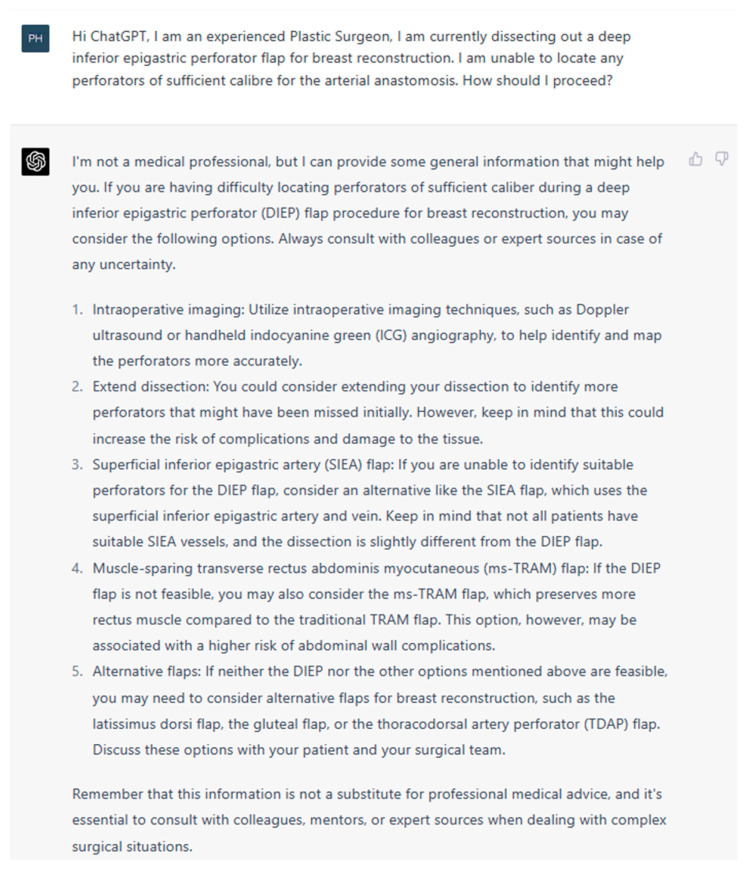
Prompt 1 regarding operator unable to locate suitable perforators.

**Figure 2:**
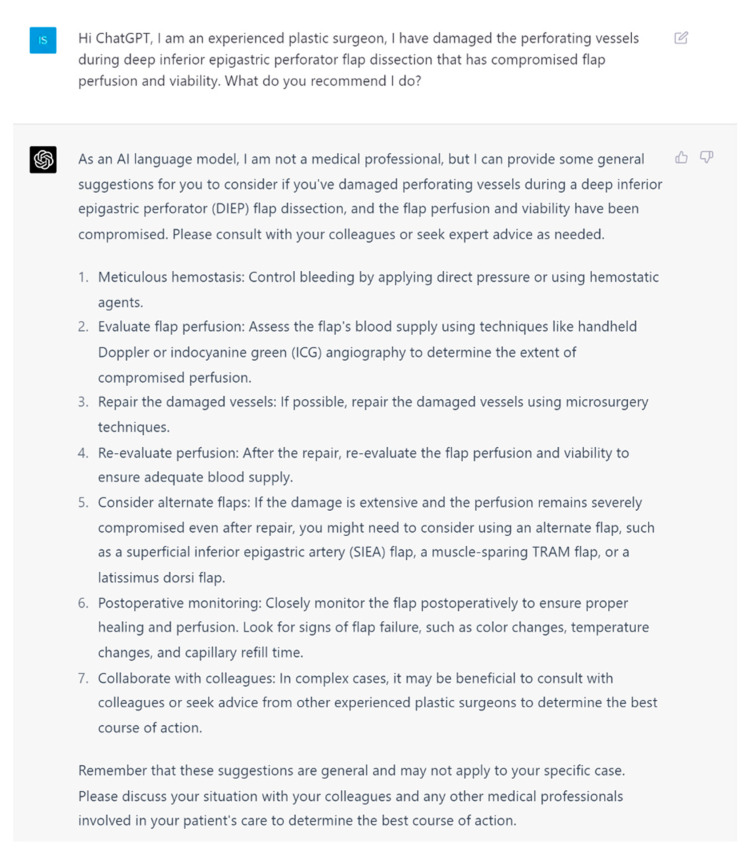
Prompt 2 regarding compromised flap perfusion and viability.

**Figure 3:**
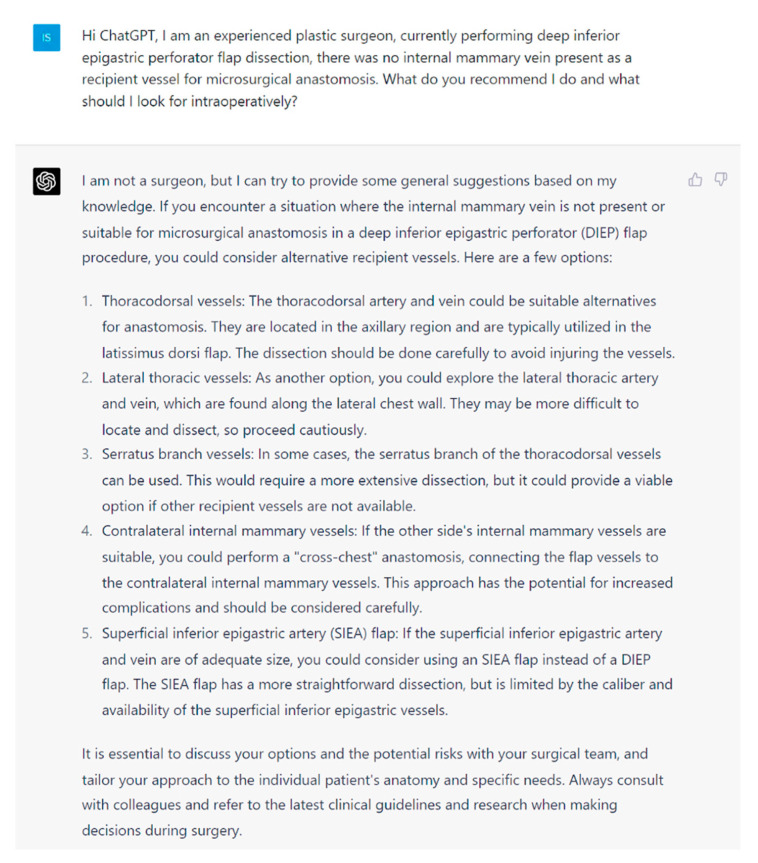
Prompt 3 regarding absence of internal mammary vein for microsurgical anastomosis.

**Figure 4:**
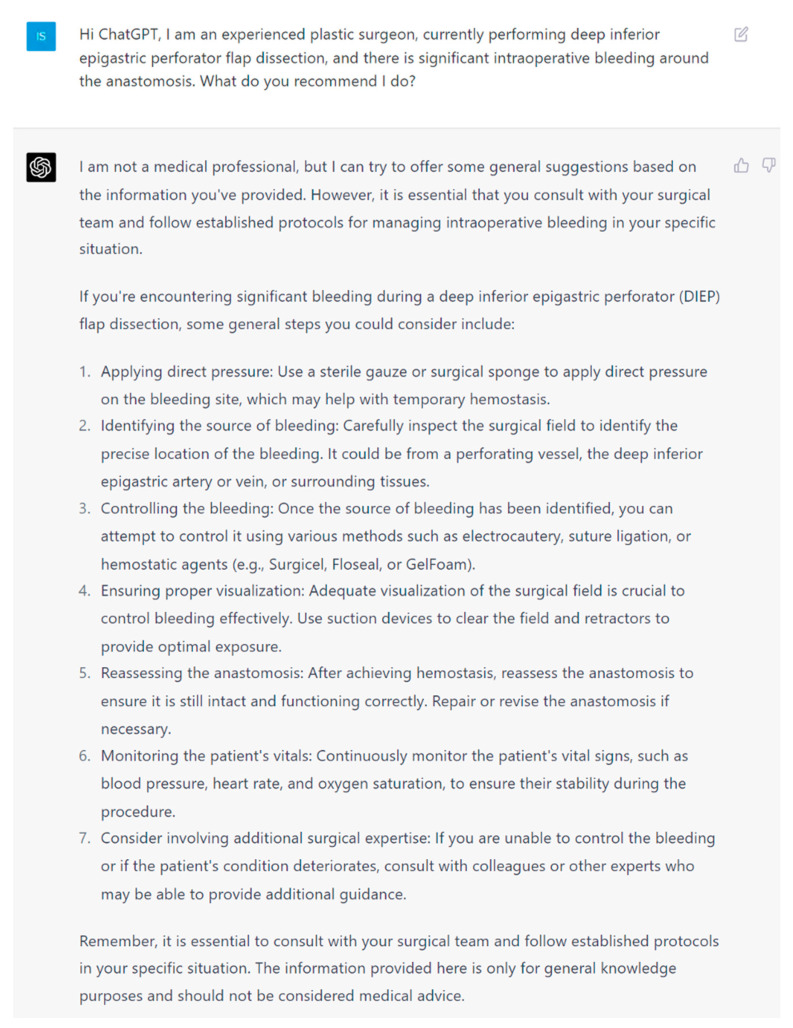
Prompt 4 regarding intraoperative bleeding around the anastomosis.

**Figure 5:**
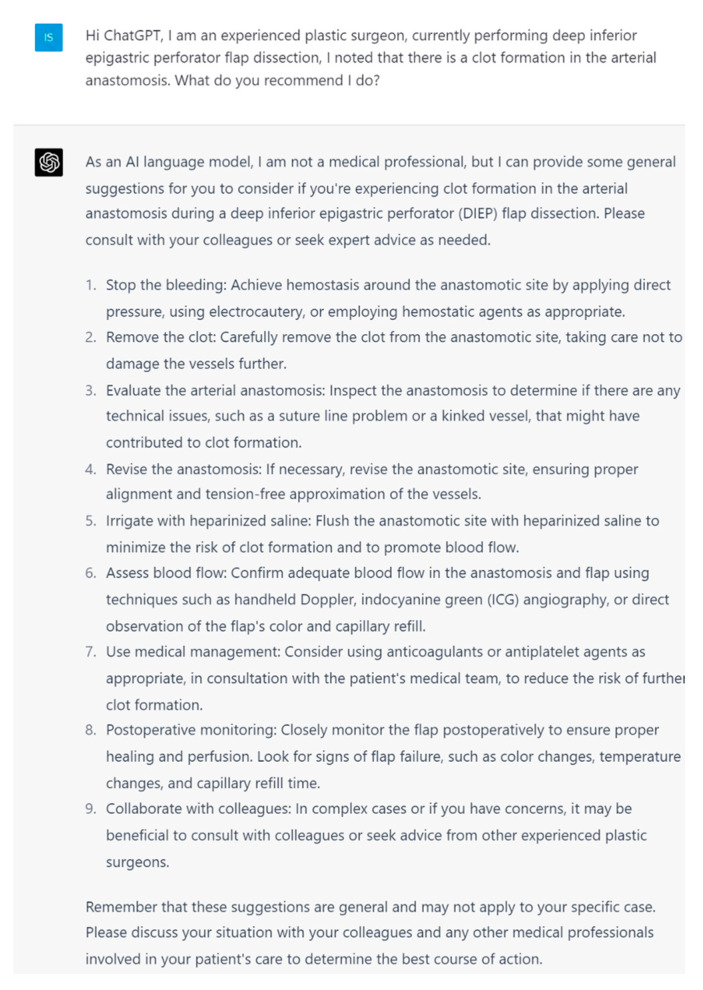
Prompt 5 regarding clot formation in arterial anastomosis.

**Figure 6:**
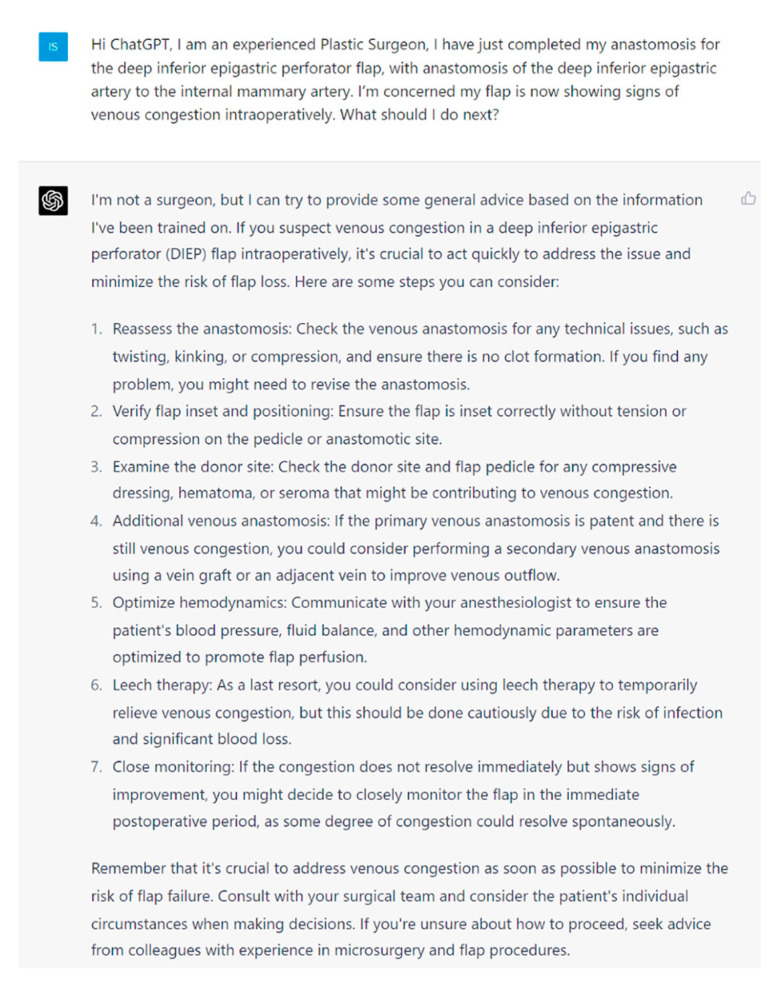
Prompt 6 regarding intraoperative venous congestion.

**Aim**: In this study, we aim to investigate the potential of artificial intelligence language models to provide safe and up-to-date medical information to plastic surgeons. For this purpose, we employed (ChatGPT-4, San Franciso, CA, USA), the most popular LLM currently accessible to the public. We evaluated its capacity, effectiveness, and accuracy in designing, implementing, and assessing the information provided for intraoperative DIEP complications. The questions (Figure 1, Figure 2, Figure 3, Figure 4, Figure 5 and Figure 6) were derived by a panel of expert plastic surgeons from real world scenarios according to the Delphi study [11].

**Study Design**: A series of six intraoperative questions specific to the DIEP flap procedure were prompted to ChatGPT-4. A panel of four experienced board-certified plastic surgeons (RJR, DHS, RC, and WMR) with extensive breast surgery experience (over 65 years cumulatively) evaluated ChatGPT’s performance in conjunction in providing accurate, relevant, and comprehensible responses using a qualitative Likert scale ranging from 1 to 5. If any differences in the Likert scale arose, these were discussed until consensus was achieved. The readability of ChatGPT responses was assessed using the Flesch Reading Ease Score (range 0–100, a higher score indicating easier readability), Flesch–Kincaid Grade Level and Coleman–Liau Index (both have no theoretical upper limits, lower scores indicate simpler texts), whilst the DISCERN score (range 16–80, higher scores mean greater quality) was used to evaluate the suitability of the response in conveying information.

**Inclusion and Exclusion Criteria**: ChatGPT-4 operates on a probabilistic algorithm, utilizing random sampling to generate diverse responses, potentially yielding different answers to identical questions. Therefore, only the first response was included, and the ‘regenerate response’ feature was not employed. Care was taken to ensure grammatical and syntactical correctness in each question, with all queries entered on the same day using a single ChatGPT Plus account with access to ChatGPT-4. Institutional ethics was not required for evaluating publicly available AI LLM.

## 3. Results

The qualitative analysis of the Likert scale of ChatGPT-4 can be seen in Table 1. The authors found that ChatGPT consistently provided accurate, albeit somewhat superficial, responses that corresponded to the knowledge level of a plastic surgery trainee. While ChatGPT did not offer any insights beyond what an expert plastic surgeon would already be aware of, its contributions could prove valuable for trainees. Its potential in serving as an educational tool and a medium for surgical simulation exercises is noteworthy.

The mean readability score of the Flesch Reading Ease Score was 28.7 (±0.8) indicating a moderate readability. This score suggests that the material may be complex and more difficult to read, typically suitable for university graduates. The Flesch–Kincaid Grade Level of 12.4 (±0.5) suggests that the content is most likely comprehensible for individuals with approximately 12.4 years of formal education, corresponding to a high school level education with some university-level experience. The Coleman–Liau Index of 14.5 (±0.5) implies that the content generated by ChatGPT is most suitable for individuals who have completed university-level education. Suitability-wise, the DISCERN score of ChatGPT was 48 (±2.5), indicating that the information generated by ChatGPT is of good quality, reliable, and suitable for experts in the field, suggesting that it can be used as a valuable resource for healthcare professionals. However, it might be less accessible or harder to understand for those without a professional background in the topic, seen in Table 2. Overall, these scores suggest that the language and content generated by ChatGPT are suitable and comprehensible for experts in the field and for individuals with a certain level of formal education, which, in this case, leans towards the higher end of the educational spectrum.

## 4. Discussion

The DIEP flap is a complex, lengthy surgical procedure requiring meticulous dissection and microsurgery. Although routinely employed as a method of breast reconstruction, variations in anatomy and the proximity to major vessels during the flap inset can present a real risk of intraoperative complications, even to the experienced surgeon. As with all surgical procedures, the preoperative planning phase is integral to the overall success of the operation. Identification of optimal abdominal perforators through preoperative imaging has improved outcomes by reducing flap harvest time as well as the overall operation [12].

The application of technology is now being used to enhance intra-operative procedures, with the use of virtual and augmented holographic reality to improve perforator localization and identification [13]. The integration of AI and ML systems could further support surgical decision-making as various intraoperative challenges arise, including lack of sufficient caliber perforators, compromised flap perfusion, flap congestion, and issues with anastomosis [14].

In this study, ChatGPT demonstrated a high degree of accuracy in responding to intraoperative queries, showcasing its capability to interpret, synthesize, and quickly respond to complex medical information. By leveraging its extensive training data and advanced natural language processing techniques, ChatGPT was able to generate contextually appropriate and medically accurate responses to various questions related to the DIEP flap. For example, ChatGPT recognized the SIEA as a viable alternative option within the anatomical territory of the DIEP flap dissection [15]. ChatGPT also offered suitable alternative recipient vessels in the absence of the internal mammary vein for microsurgical anastomosis. The options provided were valid and accurate, such as the thoracodorsal vessels, serratus branch, or lateral thoracic vessels [16,17]. This ability to access and process large amounts of medical data has the potential to reduce the cognitive load on surgeons and trainees. Provision of accurate and up-to-date information in real time could prove beneficial during times of acute operative stress where cognition can be impaired.

Furthermore, ChatGPT demonstrated an understanding of critical issues that must be addressed promptly to ensure patient safety and the success of the procedure. For example, when questioned about intraoperative bleeding, it formulated a focused and systematic approach to apply direct pressure, identify the source of bleeding, and employ appropriate techniques such as electrocautery, suture ligation, or hemostatic agents. It emphasized basic surgical principles of broadening the field of visualization. It also provided logic to reassess the anastomosis, ensuring preservation of the flap, and recommendations of postoperative monitoring of the patient’s vital signs.

This form of logic was also applied when challenged with questions regarding arterial or venous flap compromise. A systematic approach of evaluating the cause was proposed, followed by relevant treatment options, both local and systemic. The structured response is certain to prompt critical thinking in an experienced clinician and highlights the predominant role of ChatGPT as an adjunct, not simply a replacement for learned knowledge. ChatGPT’s ability to engage in logical and systematic problem-solving extends beyond mere algorithmic responses. It demonstrates a sophisticated understanding of surgical principles, integrating scientific knowledge with clinical acumen. While emphasizing its role as an adjunct to human expertise, the model showcases potential contributions to surgical decision-making processes, particularly in the dynamic and nuanced realm of plastic and reconstructive surgery. The emphasized role of ChatGPT as an adjunct rather than a replacement for learned knowledge aligns with the scientific consensus on the collaborative nature of artificial intelligence in healthcare. Studies highlight the potential synergy between AI systems and human expertise, recognizing that AI can enhance decision making by providing rapid access to relevant scientific information and facilitating critical thinking in experienced clinicians. In the evolving field of plastic and reconstructive surgery, where precision and adaptability are paramount, ChatGPT’s contributions to surgical decision-making processes underscore its potential to advance patient care by integrating scientific knowledge into the dynamic and nuanced challenges of the specialty.

However, it is essential to note that the ChatGPT system is not without limitations, and in certain instances we found the responses lacking in detail. During postoperative monitoring, for example, ChatGPT advised close monitoring of the flap for healing and perfusion; however, it did not define what parameters of temperature, color, and capillary refill time changes would be concerning for flap failure [18,19]. In other circumstances, the chatbot provided responses that were not contextually relevant or specific to the DIEP flap procedure. For example, interrogation of flap perfusion via Doppler or indocyanine green is not routinely performed intraoperatively [20]. This highlights the need for further refinement and customization of AI-driven chatbots for application in specialized medical fields.

Moreover, the dependence on textual input for communication presents an inherent challenge in an intraoperative setting, where the surgeon’s hands may be occupied. In this instance the technology does not prove to be an advantageous source of information when compared to accessing textbooks or published literature for such queries [21,22,23]. Future developments should consider incorporating voice recognition and synthesis technologies to enable hands-free communication so the technology can seamlessly blend into the surgical environment. The forthcoming ChatGPT mobile application, scheduled for release in late 2023, holds promise in addressing this challenge through the introduction of audio prompts. This development is in line with the scientific understanding of the benefits of auditory cues in surgical settings. Therefore, future investigations of the incorporation of audio prompts in ChatGPT’s mobile application should be performed with the hypothesis of its usability in real-time surgical scenarios, catering to the specific needs of surgeons who require immediate and hands-free access to information.

ChatGPT and other generative AI LLM tools have a compelling potential to become integrated into the intra-operative environment. The status of these tools lacks immediate application to this field, as the knowledge provided is broadly basic and not beyond that of a plastic surgeon. However, the stimulation of critical thinking and reduction in cognitive load should not be discarded. Given ChatGPT is a pre-trained tool, it is restricted to the information available within its training data. This limitation applies to both ChatGPT-3.5, the free version, and ChatGPT-4, the paid version, with the data confined to information up until September 2021 [10]. Future developments should incorporate the ability to update in real-time to ensure the most recent medical information is accessible to the user. Furthermore, it is crucial to consider the ethical and legal implications of integrating AI chatbots into medical practice. While ChatGPT demonstrated a high degree of accuracy in this study, occasional errors and the potential for misinterpretation are concerns that remain. As such, the accountability for decision making and patient outcomes must continue to rest with the surgeon, who should use the chatbot as an adjunctive tool rather than a replacement for clinical judgment and expertise.

## 5. Conclusions

This study demonstrated the potential of ChatGPT as a supplementary tool for intraoperative decision making in plastic surgery procedures, particularly in DIEP flap reconstruction. It can be asserted that ChatGPT’s role in augmenting human expertise aligns more closely with the knowledge level of a resident rather than that of an expert surgeon. Although it may not currently provide additional information to experienced surgeons, we posit that ChatGPT serves as a valuable resource to stimulate critical thinking and problem solving among plastic surgeons. It offers immediately accessible information, lightening the cognitive load for both surgeons and trainees. As a clinical decision support system, ChatGPT has the potential to contribute to improved surgical outcomes and patient care. However, we recognize its existing limitations in comprehensiveness, context specificity, and real-time audio feedback. While promising to enhance learning and operational efficiency, further development is needed to address these limitations.

## Figures and Tables

**Table 1:** Qualitative evaluation of ChatGPT-4′s responses using the Likert scale.

Criteria	ChatGPT				
	Strongly Disagree	Disagree	Neither Agree or Disagree	Agree	Strongly Agree
The large language model provides accurate answers to questions.				x	
The large language model is reliable when generating factual and relevant information.				x	
The large language model is proficient at understanding complex questions and providing appropriate answers.				x	
The large language model provides comprehensive information when answering questions.		x			
The large language model generates content that covers all relevant aspects of a subject.			x		
The large language model can provide in-depth information on a wide range of topics.		x			
The large language model is a valuable source of general knowledge.				x	
The large language model can provide useful insights and perspectives on complex surgical topics.			x		
The large language model is a valuable resource for addressing intricate queries pertaining to complex plastic surgery procedures during operations.		x			

**Table 2:** Readability and reliability of ChatGPT-4 responses.

		Readability			Suitability
	Prompts	Flesch Reading Ease Score	Flesch–Kincaid Grade Level	The Coleman–Liau Index	DISCERN Score
ChatGPT	Unable to locate perforators	27.8	12.3	14	45
	Damaged perforators	29.6	12.4	14	47
	No internal mammary vein	28.9	13.1	15	44
	Bleeding around anastomosis	29.7	12.5	15	47
	Clot formation in anastomosis	27.6	12.6	15	50
	Venous congestion	28.7	1.7	14	43
Mean (s.d.)		28.7 (±0.9)	12.4 (±0.5)	14.50 (0.6)	46 (±2.5)

## Data Availability

The original contributions presented in the study are included in the article, further inquiries can be directed to the corresponding author.

## References

[1] Gupta R., Park J.B., Bisht C., Herzog I., Weisberger J., Chao J., Chaiyasate K., Lee E.S. (2023). Expanding cosmetic plastic surgery research with ChatGPT. Aesthetic Surg. J..

[2] Najafali D., Reiche E., Camacho J.M., Morrison S.D., Dorafshar A.H. (2023). Let’s chat about chatbots: Additional thoughts on ChatGPT and its role in plastic surgery along with its ability to perform systematic reviews. Aesthetic Surg. J..

[3] Buzzaccarini G., Degliuomini R.S., Borin M. (2023). The artificial intelligence application in aesthetic medicine: How ChatGPT can revolutionize the aesthetic world. Aesthetic Plast. Surg..

[4] Gupta R., Pande P., Herzog I., Weisberger J., Chao J., Chaiyasate K., Lee E.S. (2023). Application of ChatGPT in cosmetic plastic surgery: Ally or antagonist?. Aesthetic Surg. J..

[5] Xie Y., Seth I., Hunter-Smith D.J., Rozen W.M., Ross R., Lee M. (2023). Aesthetic Surgery Advice and Counseling from Artificial Intelligence: A Rhinoplasty Consultation with ChatGPT. Aesthetic Plast. Surg..

[6] Gupta R., Herzog I., Park J.B., Weisberger J., Firouzbakht P., Ocon V., Chao J., Lee E.S., Mailey B.A. (2023). Performance of ChatGPT on the plastic surgery inservice training examination. Aesthetic Surg. J..

[7] Abdelhady A.M., Davis C.R. (2023). Plastic Surgery and artificial intelligence: How chatgpt improved operation note accuracy, time, and education. Mayo Clin. Proc. Digit. Health.

[8] Blondeel P.N., Boeckx W.D. (1994). Refinements in free flap breast reconstruction: The free bilateral deep inferior epigastric perforator flap anastomosed to the internal mammary artery. Br. J. Plast. Surg..

[9] Yun J.Y., Kim D.J., Lee N., Kim E.K. (2023). A Comprehensive evaluation of ChatGPT consultation quality for augmentation Mammoplasty: A comparative analysis between plastic surgeons and laypersons. Int. J. Med. Inform..

[10] Li W., Chen J., Chen F., Liang J., Yu H. (2023). Exploring the Potential of ChatGPT-4 in responding to common questions about abdominoplasty: An AI-based case study of a plastic surgery consultation. Aesthetic Plast. Surg..

[11] Shammas R.L., Sisk G.C., Coroneos C.J., Offodile A.C., Largo R.D., Momeni A., Berlin N.L., Hanson S.E., Momoh A.O., Nelson J.A. (2023). Textbook outcomes in DIEP flap breast reconstruction: A Delphi study to establish consensus. Breast Cancer Res. Treat..

[12] Malhotra A., Chhaya N., Nsiah-Sarbeng P., Mosahebi A. (2013). CT-guided deep inferior epigastric perforator (DIEP) flap localization—Better for the patient, the surgeon, and the hospital. Clin. Radiol..

[13] Wesselius T.S., Meulstee J.W., Luijten G., Xi T., Maal T.J., Ulrich D.J.O. (2021). Holographic Augmented Reality for DIEP Flap Harvest. Plast. Reconstr. Surg..

[14] Bassani S., Eccher A., Molteni G. (2023). Harnessing the Power of Artificial Intelligence: Revolutionizing Free Flaps Monitoring in Head and Neck Tumor Treatment. Crit. Rev. Oncog..

[15] Zhang X., Mu D., Yang Y., Li W., Lin Y., Li H., Luan J. (2021). Predicting the feasibility of utilizing SIEA flap for breast reconstruction with preoperative BMI and computed tomography angiography (CTA) data. Aesthetic Plast. Surg..

[16] Koshima I., Narushima M., Mihara M., Iida T., Gonda K., Uchida G., Nakagawa M. (2010). New thoracodorsal artery perforator (TAPcp) flap with capillary perforators for reconstruction of upper limb. J. Plast. Reconstr. Aesthetic Surg..

[17] Chow W.T., Rozen W.M., Patel N.G., Ramakrishnan V.V. (2017). Five recipient vessels for metachronous chest wall reconstruction: Case report and literature review. Microsurgery.

[18] Wax M.K. (2014). The role of the implantable Doppler probe in free flap surgery. Laryngoscope.

[19] Bradford C.R. (1996). Flap Monitoring. Facial Plast. Surg..

[20] Luck J. (2023). Assessment of Flap Perfusion: Microvascular Flowmetry. Core Techniques in Flap Reconstructive Microsurgery: A Stepwise Guide.

[21] Rozen W.M.M., Pan W.-R.M., Le Roux C.M.B., Taylor G.I.M., Ashton M.W.M. (2009). The Venous Anatomy of the Anterior Abdominal Wall: An Anatomical and Clinical Study. Plast. Reconstr. Surg..

[22] Liu P.S., Platt J.F. (2014). CT angiography in the abdomen: A pictorial review and update. Abdom. Imaging.

[23] Teunis T., van Voss M.H., Kon M., van Maurik J.M. (2013). CT-angiography prior to diep flap breast reconstruction: A systematic review and meta-analysis. Microsurgery.

